# Restrictive Eating, Emotional Dysregulation, and HighRisk Behaviours in an Adolescent: A Case of Diagnostic Ambiguity in CAMHS

**DOI:** 10.1192/bjo.2026.11772

**Published:** 2026-06-30

**Authors:** Rishabh Chormalle, Suneetha Siddabattuni

**Affiliations:** Lincolnshire Partnership Foundation Trust, Lincoln, United Kingdom

## Abstract

**Aims:**

To describe the clinical complexity of an adolescent presenting with severe restrictive eating, emotional dysregulation, and high-risk behaviours where the diagnosis of Anorexia Nervosa (AN) remained uncertain within Child and Adolescent Mental Health Services (CAMHS). Additional objectives include examining how social adversity and fluctuating engagement shaped the evolving formulation and management needs.

**Methods:**

A retrospective review of clinical notes, inpatient episodes, Mental Health Act assessments, and multidisciplinary documentation was undertaken. Key events were organised chronologically, including physical health parameters, episodes of self-harm, social context, and engagement with services.

**Results:**

An adolescent known to services for a few years, demonstrated early self-harm, restrictive eating, purging, and excessive exercise. Over the years, the young person (YP) experienced multiple overdoses, admissions under the Mental Health Act, nasogastric feeding, and medical instability related to low food intake.

Although YP exhibited low weight, calorie restriction, and distress related to body image, co-existing mood instability and relational stress made the diagnosis of Anorexia Nervosa uncertain throughout CAMHS involvement. Interventions included selective serotonin reuptake inhibitors, antipsychotics, crisis work, therapy and multi-agency safeguarding involvement.

Periods of stabilisation alternated with relapse and disengagement. YP was transitioned to the adult services when 18.

**Conclusion:**

This case highlights the need for flexible, formulation driven care where restrictive eating coexists with high risk behaviours. Diagnostic ambiguity should not delay intervention, instead, consistent relational work, coordinated safeguarding, and careful transition planning are essential to support recovery.

